# Association between Dietary Carotenoid Intake and Bone Mineral Density in Korean Adults Aged 30–75 Years Using Data from the Fourth and Fifth Korean National Health and Nutrition Examination Surveys (2008–2011)

**DOI:** 10.3390/nu9091025

**Published:** 2017-09-16

**Authors:** Gebereamanuel Meron Regu, Hyesook Kim, You Jin Kim, Ju Eun Paek, Gunjeong Lee, Namsoo Chang, Oran Kwon

**Affiliations:** 1 Department of Nutritional Science and Food Management, Ewha Womans University, 52, Ewhayeodae-gil, Seodaemun-gu, Seoul 03760, Korea; meronregu@gmail.com (G.M.R.); khs7882@hanmail.net (H.K.); eugene841226@gmail.com (Y.J.K.); ca_june@hanmail.net (J.E.P.); nschang@ewha.ac.kr (N.C.); 2 Department of Global Health and Nursing, Ewha Womans University, 52, Ewhayeodae-gil, Seodaemun-gu, Seoul 03760, Korea; gunjeong@ewha.ac.kr

**Keywords:** β-carotene, β-cryptoxanthin, bone mineral density, postmenopausal female

## Abstract

Age-related bone loss is a major public health problem. This cross-sectional study examined the association between the dietary intake of carotenoids and bone mineral density (BMD). Data from 8022 subjects (3763 males and 4259 females) aged 30–75 years included in the Korean National Health and Nutrition Examination Survey (2008–2011) were analyzed. BMD was measured by dual-energy X-ray absorptiometry. Intake of carotenoids was estimated using 24-h dietary recall. In multiple linear analysis, after adjusting for covariates, lutein + zeaxanthin and β-cryptoxanthin intake was positively associated with total hip BMD in males and premenopausal women respectively, while β-carotene intake was positively correlated with femoral neck, total hip, and whole-body BMD in postmenopausal women. Postmenopausal women in the highest quintile of daily β-carotene intake, showed a lower risk of osteopenia at the lumbar spine (odds ratio (OR): 0.35, 95% CI: 0.16–0.79, *P* for trend = 0.009) than those in the lowest quintile, after adjusting for covariates. Daily β-cryptoxanthin intake was significantly associated with a lower risk of osteopenia at the total hip (OR per 1 mg/day increase: 0.76; 95% CI: 0.59–0.97), and lumbar spine (OR per 1 mg/day increase: 0.79; 95% CI: 0.70–0.89) in postmenopausal women. These results suggest that the dietary intake of β-carotene and β-cryptoxanthin may have a positive effect on bone health.

## 1. Introduction

Age-related bone loss is widely recognized as a major public health problem. A decrease in bone mineralization, which is termed osteopenia, results from the disproportion of bone resorption and bone mineralization. This can further advance to osteoporosis, which is characterized by a low bone mass and deterioration of bone tissue architecture [1]. Bone loss that appears with aging is the primary cause of osteoporotic fracture [2]. Due to the rapid expansion of the elderly population in Asia, it is expected that 45% of world hip fractures will occur in Asia by the year 2050 [3]. South Korea is one of the most rapidly aging countries in the world [4]. According to a nationwide survey undertaken from 2008 to 2011 [5,6], nearly half of Korean people aged ≥50 years have osteopenia (46.7% in women; 47.2% in men), while 38% of women and 7.3% of men aged ≥50 years have osteoporosis. Given that the prevalence of osteopenia and osteoporosis in the rapidly aging Korea society is expected to increase, it is important to develop and implement nutritional approaches and policies to prevent and treat bone mineral loss.

Bone mineral density (BMD) is associated with various lifestyle factors, such as physical activity, smoking, diet, and alcohol consumption [7,8]. Among various dietary factors, minerals [9,10,11], including calcium and vitamin D, antioxidant vitamins (vitamins C and E), flavonoids [12,13,14], and dietary patterns rich in milk and dairy products, green tea, and fruits and vegetables [15,16,17,18] have effects on bone metabolism in both younger and older age groups. Recently, some cross-sectional [19,20] and longitudinal [21,22] studies have demonstrated that antioxidant carotenoids, abundant in fruits and vegetables [23], are beneficial for the maintenance of normal bone metabolism in post-menopausal women (30–70 years), men and women (2–62 years), and also men (4–74 years). Several potential mechanisms have been proposed to explain the correlation between carotenoids and bone health, including an inhibitory effect of carotenoids on osteoclastic bone resorption related to their antioxidant activity [24,25] and their stimulatory effect on osteoblastic bone formation [26].

Previous human studies on the association between carotenoid intake and bone health have been performed in Western countries, including America [21], Australia [27], and Japan [28]. To the best of our knowledge, only one study has examined the association between dietary carotenoid intake and bone health in Korea [29]. That study reported a positive association between β-carotene intake and BMD in Korean postmenopausal women aged 50–75 years [29]. However, that study was conducted with a small sample size (*n* = 189) and a convenience sampling method.

As mentioned above, South Korea has a rapidly aging population, and bone loss is becoming a major public health problem. Thus, more research on the association between dietary intake and BMD is needed in the Korean population. Carotenoids might be a candidate that fulfills the future strategic plan for bone loss prevention with aging. Therefore, the purpose of this study was to evaluate the association between the dietary intake of carotenoids and BMD in men, and pre- and postmenopausal women aged between 30 and 75 years, using data from the Korean National Health and Nutrition Examination Survey (KNHANES) 2008–2011.

## 2. Methods

### 2.1. Study Design and Participants

In this study, data from the KNHANES 2008–2011 were analyzed. KNHANES is a national survey conducted by the Korea Centers for Disease Control and Prevention (KCDC) since 1998, to examine the general health and nutrition status of the Korean population. The KNHANES uses a stratified, multistage, and clustered probability sampling design for the selection of household units. It consists of a health interview survey, health examination survey, and nutrition survey. The sampling weights for each sample individual are the product of three factors; the reciprocal of the probabilities of selection (primary selection unit, household); an adjustment for non-response (household); and a post-stratification factor to make the resulting survey estimates for age, gender, metropolitan area, or province category approximately equal to the total population of Korea. Thus, the calculated estimates are an accurate representation of the Korean population. The fourth (2008–2009) and fifth (2010–2011) KNHANES were conducted throughout the year to avoid seasonal bias in the diet. BMD measurements were first included in the second year (2008) of the KNHANES IV.

This study was approved by the Institutional Review Board of the Korea Centers for Disease Control and Prevention (2008-04EXP-01-C, 2009-01CON-03-2C, 2010-02CON-21-C, and 2011-02CON-06-C). Written informed consent was obtained from all subjects. Detailed information about the survey is available at http://knhanes.cdc.go.kr. A total of 37,753 participants completed the survey between 2008 and 2011.

**Figure 1:**
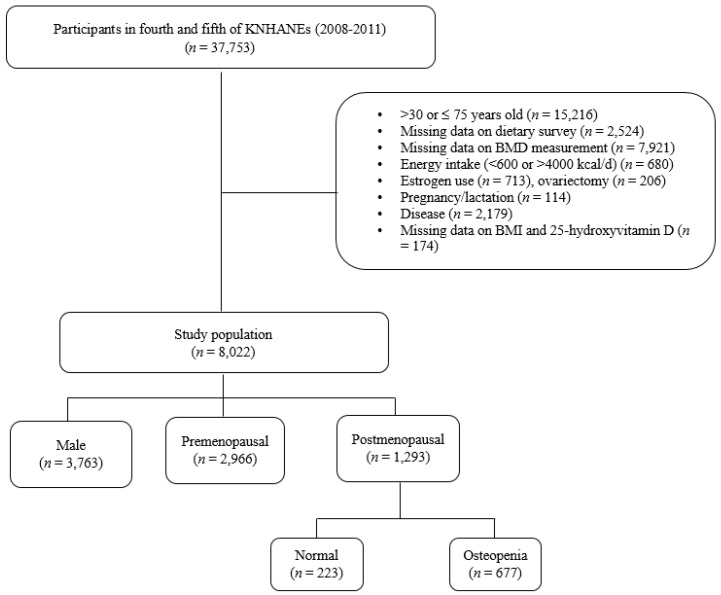
Flow chart of the subject inclusion and exclusion criteria in the Korean National Health and Nutrition Examination Survey (KNHANES) 2008–2011.

Subjects aged <30 or ≥75 years (*n* = 15,216) were excluded. The following participants were also excluded: those who did not respond to the dietary survey (*n* = 2524), had missing data for dual-energy X-ray absorptiometry (DXA) examination (*n* = 7921), had an energy intake < 600 or >4000 kcal/day (*n* = 680), used estrogen (*n* = 713), had ovariectomy (*n* = 206), were pregnant or lactating (*n* = 114), had bone metabolism-related diseases (such as arthritis, bone arthritis, and rheumatism), renal failure, thyroid dysfunction, cancer, or hepatitis (types B and C) (*n* = 2179), had missing data for body mass index (BMI) and serum 25-hydroxyvitamin D level (25(OH)D; *n* = 174), or had missing data for menopausal status (*n* = 4). Finally, 8022 subjects were included and stratified by gender and menopausal status, including 3763 males, 2996 premenopausal women, and 1293 postmenopausal women (Figure 1).

### 2.2. Measurements of Anthropometric Parameters and BMD

Anthropometric measurements were taken by well-trained examiners. Height and weight (in light clothes) were measured by standard methods. Height was measured to the nearest 0.1 cm, and weight was measured to the nearest 0.1 kg. BMI was calculated as the ratio of weight (kg) to height (m^2^). BMD (g/cm^2^) measurements were obtained using dual-energy X-ray absorptiometry (DXA, Discovery. QDR 45000; Hologic Inc., Waltham, MA, USA). The DXA scanner was calibrated daily, using a spine phantom and weekly using a step phantom. The DXA results were reviewed and analyzed at the Korean Society of Osteoporosis (Seoul, Korea), using industry-standard techniques. The analysis was performed using Hologic Discovery software (version 13.1 Hologic, Inc., Waitham, MA, USA). Diagnosis of osteopenia was made using the World Health Organization (WHO) T-score criteria (−2.5 < T-score < −1.0) for Asians. Serum 25(OH)D level was measured using a gamma counter (1470 Wizard; Perkin Elmer, Turku, Finland) with a radioimmunoassay (RIA) kit (DiaSorin Inc., Stillwater, MN, USA). The serum 25(OH)D inter-assay coefficients of variation were 19.6–6.1% for the samples [30]. The measurement of 25(OH)D was standardized according to the measurement procedures of the National Institute of Standards and Technology (NIST) and the Ghent University Vitamin D Standardization Program (VDSP).

### 2.3. Lifestyle Questionnaires

Participants completed a face-to-face interview, containing 133 standardized health questions that consisted of household and individual components. The household data were provided by an adult respondent aged ≥19 years and included variables on demographics. The individual components included information on cigarette smoking, alcohol use, physical activity, and mental health. Smoking status was categorized as a non-smoker and current smoker. Alcohol consumption was categorized as non-drinker and current drinker. Physical activity was assessed with the international physical activity questionnaire (IPAQ) and categorized as “yes” or “no”. Subjects who exercised vigorously for >20 min at least three times a week or moderate exercise or walking for >30 min at least five times a week were considered as doing regular exercise or “yes” subjects. Educational levels of participants were divided into elementary or lower school, middle school, high school, and college or above. A dietary supplement was considered a product consumed to supplement the diet, such as functional foods and included all kinds of supplements. The respondents were asked to reply ‘yes’ or ‘no’, to whether they had used a supplement for more than two weeks during the last year.

### 2.4. Dietary Assessment and Carotenoids Database

Dietary intakes were assessed by 24-h dietary recalls. Data were collected from each participant by dietary interviewers trained by the KCDC. Daily carotenoid intake was estimated by merging individual food items from the KNHANES with the United States Department of Agriculture (USDA)–Nutrition Coordinating Center carotenoid database that includes 2326 food items [31].

### 2.5. Statistical Analysis

Carotenoid and nutrient intake were adjusted for total energy intake using the residual method [32]. Logarithmic transformation was applied to achieve normality before creating residuals. All subjects were categorized into three groups stratified by gender and menopausal status. The distribution of general characteristics in each group was analyzed using the PROC SURVEYFREQ procedure. The crude weight mean and standard error of continuous variables were analyzed by PROC SURVEYMEANS procedure.

The potential confounders of the continuous variables were determined by using linear regression (LR) analysis of carotenoid intake with anthropometric and other nutrient intake variables. The same was performed for BMD with anthropometric and other nutrient intake variables. Similarly, potential confounders of the categorical variables were determined by using general linear model (GLM) analysis of carotenoid intake with lifestyle variables and supplement use. The same was applied for BMD with lifestyle variables and supplement use. Variables that showed significant linear trends in both LR and GLM analysis were considered as potential confounders. Variance inflation factor (VIF) was applied for each linear trend analysis to avoid the multicollinearity effect in the statistical models.

PROC SURVEYREG analysis was used to calculate regression coefficients (β), enabling estimated differences in BMD associated with a 1 mg increase in the intake of each type of carotenoid per day. Age, BMI, and energy-adjusted intakes of five individual carotenoids were adjusted for confounders in Model 1. Smoking behavior, alcohol consumption, physical activity, education level, supplement use, energy-adjusted intakes of fiber, vitamin C, calcium, and sodium, and serum 25(OH)D level were adjusted for confounders in Model 2. The subjects were grouped into five categories based on their carotenoid intake. Then, PROC SURVEYLOGISTIC analysis was performed, to estimate the odds ratios (ORs) and 95% confidence intervals (CIs) for osteopenia across the quantiles of carotenoid intake, where the lowest quantile was set as the reference. SAS software (version 9.4, SAS Institute Inc., Cary, NC, USA) was used for all statistical analyses. All *p*-values < 0.05 were considered statistically significant.

## 3. Results

### 3.1. Characteristics of the Study Population

The general characteristics of the subjects included in this study (Table 1) were not significantly different from those of excluded subjects (data not shown). Data for age, anthropometric measurements, lifestyle characteristics, and nutrient intake are shown in Table 1. The mean ages of male, premenopausal women, and postmenopausal women were 45.9 ± 0.2, 40.0 ± 0.1, and 58.2 ± 0.3 years, respectively.

### 3.2. Association of Carotenoid Intake with BMD

In multiple linear regression analysis, intake of lutein + zeaxanthin was positively associated with total hip BMD (β = 0.0015, *p =* 0.032) in male subjects, after adjusting for all potential covariates (Model 2) (Table 2). For premenopausal women, β-cryptoxanthin intake was positively associated with total hip BMD (β = 0.0032, *p =* 0.026), and in postmenopausal women, β-carotene intake was positively associated with femur neck BMD (β = 0.0012, *p =* 0.035), total hip BMD (β = 0.0012, *p =* 0.036), and whole-body BMD (β = 0.0021, *p =* 0.021), after adjusting for potential covariates (Model 2) (Table 2).

### 3.3. Relation between Carotenoids Intake and Risk of Osteopenia in Postmenopausal Women

In postmenopausal women after adjusting for covariates, the risk of osteopenia at the lumbar spine was 65% lower (OR: 0.35; 95% CI: 0.16–0.79, *P* for trend = 0.009) and osteopenia was also 63% lower (OR: 0.37; 95% CI: 0.15, 0.93, *P* for trend = 0.025) in the highest quintile compared with the lowest quintile of β-carotene intake (Table 3). Daily β-cryptoxanthin intake was significantly associated with a lower risk of osteopenia at total hip (24%) (OR per 1 mg/day increase: 0.76; 95% CI: 0.59–0.97), lumbar spine (21%) (OR per 1 mg/day increase: 0.79; 95% CI: 0.70–0.89), and osteopenia (12%) (OR per 1 mg/day increase: 0.88; 95% CI: 0.78–0.99) in postmenopausal women, respectively. Using 3 mg/day as a cutoff value for the intake of β-cryptoxanthin necessary to prevent osteoporosis [33], the highest cutoff value category showed a low risk of osteopenia at total hip (93%) (OR: 0.07; 95% CI: 0.01–0.68) and lumbar spine (64%) (OR: 0.36; 95% CI: 0.18–0.73) for postmenopausal women (data not shown).

## 4. Discussion

The results of this study revealed a positive association between lutein + zeaxanthin and β-cryptoxanthin intake and BMD at the total hip in males and premenopausal women, respectively. This study also demonstrated a positive association between β-carotene intake and BMD at femur neck, total hip, and whole body in postmenopausal women. Postmenopausal women in the highest quintile of daily β-carotene intake showed a decreased risk of osteopenia at the lumbar spine compared to those in the lowest quintile. Intake of each additional 1 mg/day of β-cryptoxanthin resulted in a decreased risk of osteopenia at the total hip (24%), and lumbar spine (21%), in postmenopausal women. These positive associations of β-carotene and β-cryptoxanthin with BMD concurred with previous studies [19,20,27,29,34,35]. A subpopulation cross-sectional study has demonstrated that dietary β-carotene intake is positively associated with bone mass at the lumbar spine in Australian postmenopausal women [27]. A hospital-based cross-sectional study has found a positive association between β-carotene intake and T-score at the lumbar spine in Korean postmenopausal women [29]. A case–control study in Utah has reported that (former and current) smokers in the highest quintile of β-carotene intake have 61% lower risk of hip fracture compared to the lowest quintile, whereas, in non-smokers (who have never smoked), no association was found in men and women aged ≥50 years [34]. Serum β-carotene and β-cryptoxanthin levels have also shown weak but positive associations with radial BMD in postmenopausal Japanese women [19]. A four-year follow-up Japanese study of postmenopausal women aged 30–70 years has demonstrated that those in the highest tertiles of serum β-carotene and β-cryptoxanthin levels have a lower risk (76% and 93%, respectively) of osteoporosis than those in the lowest tertiles [20]. A case–control study has revealed that serum lycopene and β-cryptoxanthin concentrations are lower in osteoporotic postmenopausal women than those in the control group [35]. Despite the evidently positive relation between β-cryptoxanthin intake and BMD discussed above, a Framingham osteoporosis study has found no cross-sectional or longitudinal association between daily β-cryptoxanthin intake and BMD in men or women aged 28–62 years [21].

The mechanism involved in the protective effect of β-carotene and β-cryptoxanthin on bone mass has been investigated in several studies both in vivo [26,36,37] and in vitro [24,25,38]. Several studies have suggested that β-carotene [25] and β-cryptoxanthin [26] may have an inhibitory effect on bone resorption, due to their antioxidative activities. Wang et al., reported that β-carotene can suppress osteoclastogenesis and bone resorption, by suppressing the receptor activator of nuclear factor kappa-B ligand (RANKL) signaling pathway [25]. Some mechanistic studies have revealed that β-cryptoxanthin may have an inhibitory effect on osteoclastic bone resorption, through reducing bone resorption factors, such as parathyroid hormone and prostaglandin E2 [26] or reducing stimulatory proteins in the RANKL signaling pathway [37]. β-cryptoxanthin may also have a stimulatory effect on bone formation, by increasing the alkaline phosphate activity and the expression level of Runt-related transcriptional factor 2 (Runx2) [24,39,40] in osteoblastic cells.

In our study, a positive association between lutein + zeaxanthin intake and BMD at total hip was observed in men, but not in women. Sahni et al., have reported that the protective effect of carotenoid intake (total carotenoids, β-carotene, lycopene, and lutein + zeaxanthin) against four-year loss in trochanter BMD, is significant for men [21]. The protective effect of lycopene intake against four-year loss in lumbar spine BMD is also significant in women [21]. Biological differences between males and females, such as sex hormones and bone metabolism [41], and differences in food consumption between men and women [42], might have contributed to such gender differences in the association between carotenoid intake and BMD. Although smoking status was considered as a covariate in the present study, some residuals of factors might have resulted in the non-significant relationship between β-carotene or β-cryptoxanthin intake and BMD in males. It has been reported that smoking and alcohol drinking are significantly associated with a lower dietary intake and plasma or serum concentrations of α-carotene, β-carotene, and β-cryptoxanthin [43,44].

Our results also revealed that the risk for osteopenia at the lumbar spine was 65% lower in the highest quintile compared with the lowest quintile of dietary β-carotene intake in postmenopausal women. This association did not appear in premenopausal women. Considering that total hip and lumbar spine are highly susceptible to postmenopausal osteoporosis-related fractures [45], this finding in postmenopausal women may be meaningful and helpful for preventing and treating osteopenia. Although the exact reason why this association differs according to menopausal status is currently unknown, the sharp decrease in estrogen level during menopause might be related to such a discrepancy. Differences in β-carotene intake between pre- and postmenopausal women might have also contributed to such results. The β-carotene intake was the highest in our postmenopausal subjects among the intakes of carotenoids, and it was higher than the premenopausal subjects. The food sources considered high in β-carotene concentration were sweet potato, followed by carrot, and kale. Based on our result that a minimum 7.20 mg/day (the highest quintile value) of β-carotene consumption has a positive impact on lowering the risk of osteopenia, at least 85 g/day sweet potato, 87 g/day carrot, and 121 g/day kale consumption might fit the required amount to lower the risk of osteopenia. For β-cryptoxanthin, persimmon was the best source, followed by papaya and paprika. For lutein + zeaxanthin, dandelion was the main food source, followed by spinach and sweet potato.

In Korean postmenopausal women, the daily mean β-carotene intake was 5.16 mg/day, which was higher than that in Japan (1.86 mg/day) [28], Singapore (2.19 mg/day) [22], and the USA (3.89 mg/day) [21]. However, it was lower than that reported in Australia (6.07 mg/day) [46]. The mean intake of β-cryptoxanthin (0.68 mg/day) in this study was higher than that reported in the USA (0.06 mg/day) [21], Singapore (0.24 mg/day) [22], Australia (0.30 mg/day) [46], and Japan (0.62 mg/day) [28]. In Korean male subjects, the daily mean intake of lutein + zeaxanthin was 1.73 mg/day, which was higher than that reported in Australia (0.85 mg/day) [46], but lower than Singapore (1.85 mg/day) [22], Brazil (2.30 mg/day) [47], and the USA (2.80 mg/day) [21]. This implies that Korean postmenopausal women might have a relatively high carotenoid (eq. β-carotene, β-cryptoxanthin) intake compared to women in other countries. However, Korean men might have a lutein + zeaxanthin intake comparable to males in other countries.

This study had some limitations. First, the results in this study were obtained from a cross-sectional analysis, limiting this approach to a cause–effect relationship. Second, due to intra-individual variability in food and nutrient intakes of most people, a single 24-h dietary recall might not be sufficient, to estimate daily carotenoid intake. Third, the carotenoid contents of food can differ, depending on the climate, cultivation, harvesting, and processing conditions of plants and their storage conditions. These factors might lead to weakened estimates of effect size. Fourth, some medications that affect bone health (e.g., bisphosphonates, aromatase inhibitors, gonadotropin-releasing hormone analogs or luteinizing hormone releasing agonists, antiandrogens, and Paget’s disease treatment) were not controlled for confounders. However, this study has some strengths. First, the subjects used in this study represented the Korean population. Additionally, this is the first study that shows a positive association between individual carotenoid intake and bone health in the Korean population. Currently, South Korea has a rapidly aging population. Bone health including osteopenia is becoming a major public health problem in South Korea. The results of this study can provide valuable information for the further development of nutritional strategies and education for Korean people to improve their bone health.

## 5. Conclusions

There are significant and positive associations between β-carotene or β-cryptoxanthin intake and bone health in postmenopausal women. Lutein + zeaxanthin and β-cryptoxanthin is also positively associated with BMD in males and premenopausal women. These results suggest that increasing the intake of foods rich in β-carotene, β-cryptoxanthin, and lutein + zeaxanthin may provide benefits to bone health. Further cohort or intervention studies are needed, to confirm these findings.

## Figures and Tables

**Table 1:** Characteristics of male, premenopausal, and postmenopausal subjects ^1^.

	Male	Female		*p*-Value
		Pre-Menopausal	Post-Menopausal	
*n*	3763	2966	1293	
Age (years)	45.9 ± 0.2	40.0 ± 0.1	58.2 ± 0.3	0.339
30–39	992 (26.4)	1507 (50.8)	5 (0.4)	
40–49	980 (26.0)	1266 (42.7)	88 (6.8)	
50–59	858 (22.8)	193 (9.5)	576 (44.6)	
60–69	691 (18.4)		458 (35.4)	
70–75	242 (6.4)		166 (12.8)	
Height (cm)	170.2 ± 0.1	158.7 ± 0.1	154.4 ± 0.2	<0.0001
Weight (kg)	70.2 ± 0.2	57.7 ± 0.2	56.8 ± 0.2	<0.0001
BMI (kg/cm^2^)	24.2 ± 0.1	22.9 ± 0.1	23.8 ± 0.1	<0.0001
Waist circumference (cm)	84.6 ± 0.2	76.1 ± 0.2	81.0 ± 0.3	<0.0001
Education				<0.0001
Elementary or lower	515 (11.9)	126 (5.2)	655 (53.8)	
Middle school	431 (11.6)	203 (8.3)	173 (17.7)	
High school	1154 (36.8)	1318 (49.6)	223 (22.6)	
College or higher	1192 (39.6)	995 (37.0)	61 (5.9)	
Current smoker	1631 (47.0)	150 (5.6)	51 (4.6)	<0.0001
Current drinker	2854 (77.6)	1457 (49.2)	353 (30.0)	<0.0001
Regular exercise	2019 (53.0)	1464 (50.0)	709 (55.3)	0.008
Supplement use	1310 (34.0)	1348 (44.7)	612 (48.2)	
Nutrient intake				
Total energy (kcal/day)	2289 ± 13.9	1705 ± 12.9	1607.9 ± 20.8	<0.0001
Fiber (g/day)	7.1 ± 0.1	7.4 ± 0.1	8.1 ± 0.2	<0.0001
Vitamin C (mg/day)	95.5 ± 1.4	110.9 ± 1.8	111.9 ± 2.8	<0.0001
Calcium (mg/day)	478.3 ± 4.7	499.7 ± 5.8	476.0 ± 8.4	0.028
Sodium (mg/day)	4958.5 ± 45.9	4839.7 ± 54.2	4641.3 ± 93.1	0.005
Total carotenoids (mg/day)	6.7150 ± 0.15	9.6691 ± 0.27	10.5751 ± 0.45	<0.0001
α-Carotene (mg/day)	0.6163 ± 0.03	0.8571 ± 0.04	1.0455 ± 0.08	<0.0001
β-Carotene (mg/day)	3.0809 ± 0.08	4.5939 ± 0.16	5.0969 ± 0.23	<0.0001
β-Cryptoxanthin (mg/day)	0.2536 ± 0.02	0.5454 ± 0.04	0.6136 ± 0.06	<0.0001
Lutein + zeaxanthin (mg/day)	1.7416 ± 0.07	2.1661 ± 0.08	2.5890 ± 0.16	<0.0001
Lycopene (mg/day)	0.8190 ± 0.07	2.2920 ± 0.24	1.9692 ± 0.36	<0.0001
Bone mineral density				
Femur neck BMD (g/cm^2^)	0.8135 ± 0.02	0.7593 ± 0.03	0.6486 ± 0.03	<0.0001
Total hip BMD (g/cm^2^)	0.9733 ± 0.02	0.9025 ± 0.03	0.8033 ± 0.04	<0.0001
Lumbar spine BMD (g/cm^2^)	0.9693 ± 0.03	0.9957 ± 0.03	0.8192 ± 0.05	<0.0001
Whole body BMD (g/cm^2^)	1.1887 ± 0.04	1.1477 ± 0.03	1.0210 ± 0.04	<0.0001
Serum 25(OH)D (ng/mL)	19.2 ± 0.2	16.2 ± 0.2	18.1 ± 0.3	<0.0001

^1^ Data are shown as mean ± standard error, *n* (%). BMI, body mass index; BMD, bone mineral density; 25(OH)D, serum 25-hydroxyvitamin D. One-way analysis of variance (ANOVA) test for continuous variable and chi-square test for categorical variables used to compare the three groups. All carotenoid and nutrient intake presented as energy-adjusted values. Total carotenoid intake: the sum of the intake of five individual carotenoids.

**Table 2:** Multiple linear regression analysis for the association between BMD and daily carotenoid intake in male, and pre- and post-menopausal female subjects ^1^.

Variable	Male					Pre-Menopausal Female				Post-Menopausal Female		
	Model 1		Model 2		Model 1		Model 2		Model 1		Model 2	
	β	*P*	β	*P*	β	*P*	β	*P*	β	*P*	β	*P*
Femur neck BMD (g/cm^2^)												
α-Carotene (mg/day)	0.0020	0.335	−0.0029	0.157	0.0003	0.902	−0.0018	0.412	−0.0018	0.097	−0.0017	0.119
β-Carotene (mg/day)	0.0006	0.350	−0.0001	0.880	0.0008	0.081	0.0005	0.255	0.0014	0.002	0.0012	0.035
β-Cryptoxanthin (mg/day)	0.0002	0.936	−0.0023	0.407	0.0031	0.022	0.0022	0.116	−0.0011	0.575	0.0001	0.950
Lutein + zeaxanthin (mg/day)	0.0010	0.209	0.0013	0.080	−0.0001	0.828	0.0001	0.988	0.0008	0.364	0.0004	0.656
Lycopene (mg/day)	0.0005	0.317	−0.0004	0.432	0.0003	0.241	0.0001	0.858	0.0005	0.180	0.0001	0.704
Total hip BMD (g/cm^2^)												
α-Carotene (mg/day)	0.0027	0.197	−0.0020	0.350	0.0002	0.933	−0.0018	0.399	−0.0017	0.174	−0.0017	0.162
β-Carotene (mg/day)	0.0004	0.586	−0.0005	0.447	0.0008	0.131	0.0004	0.348	0.0015	0.003	0.0012	0.036
β-Cryptoxanthin (mg/day)	0.0029	0.327	0.0008	0.789	0.0044	0.001	0.0032	0.026	0.0002	0.922	0.0015	0.486
Lutein + zeaxanthin (mg/day)	0.0009	0.249	0.0015	0.032	0.0002	0.784	0.0001	0.816	0.0008	0.341	0.0006	0.492
Lycopene (mg/day)	0.0006	0.232	−0.0003	0.502	0.0002	0.295	−0.0003	0.233	0.0006	0.140	0.0001	0.746
Lumbar spine BMD (g/cm^2^)												
α-Carotene (mg/day)	0.0015	0.554	−0.0026	0.298	0.0010	0.704	−0.0012	0.587	−0.0027	0.090	−0.0019	0.199
β-Carotene (mg/day)	0.0008	0.234	−0.0002	0.781	0.0007	0.207	0.0001	0.927	0.0019	0.006	0.0009	0.211
β-Cryptoxanthin (mg/day)	0.0015	0.691	−0.0009	0.823	0.0002	0.924	0.0001	0.962	−0.0007	0.832	0.0010	0.720
Lutein + zeaxanthin (mg/day)	0.0009	0.261	0.0011	0.203	0.0005	0.575	0.0005	0.504	0.0015	0.130	0.0007	0.486
Lycopene (mg/day)	0.0002	0.746	−0.0007	0.283	0.0008	0.022	0.0001	0.942	0.0001	0.964	−0.0006	0.249
Whole body BMD (g/cm^2^)												
α-Carotene (mg/day)	0.0057	0.015	0.0006	0.777	0.0020	0.455	−0.0005	0.828	−0.0016	0.346	−0.0016	0.322
β-Carotene (mg/day)	0.0009	0.222	0.0001	0.998	0.0005	0.271	−0.0002	0.671	0.0027	<0.0001	0.0021	0.027
β-Cryptoxanthin (mg/day)	−0.0015	0.651	−0.0028	0.390	−0.0008	0.688	−0.0017	0.362	−0.0035	0.274	−0.0008	0.796
Lutein + zeaxanthin (mg/day)	0.0004	0.657	0.0006	0.534	0.0007	0.469	0.0006	0.508	0.0008	0.394	0.0001	0.895
Lycopene (mg/day)	0.0006	0.373	−0.0002	0.739	0.0006	0.047	−0.0003	0.285	0.0003	0.685	−0.0002	0.560

^1^ Standard regression coefficients of the bone mineral density with daily carotenoid intake were calculated by multiple linear regression analysis after adjusting for covariates. BMD, bone mineral density. Model 1: adjusted for age and BMI. Model 2: adjusted for age, BMI, alcohol consumption, physical activity, education level, supplement use, energy-adjusted intake of fiber, vitamin C, calcium and sodium, serum 25(OH)D in pre- and postmenopausal female subjects, and smoking behavior in male subjects. BMI: body mass index.

**Table 3:** Odds ratios and 95% confidence intervals of carotenoid intake on osteopenia in postmenopausal female subjects ^1^.

	Range of Carotenoid Intake	Femur Neck Osteopenia		Total Hip Osteopenia		Lumbar Spine Osteopenia		Osteopenia	
		Model 1	Model 2	Model 1	Model 2	Model 1	Model 2	Model 1	Model 2
α-Carotene (mg/day)									
Continuous (1 mg/day intake increment)		1.01 (0.93, 1.09)	0.99 (0.90, 1.08)	1.02 (0.95, 1.11)	1.04 (0.95, 1.14)	1.04 (0.95, 1.15)	1.04 (0.93, 1.16)	1.06 (0.96, 1.16)	1.05 (0.94, 1.17)
Q1	(0.0001, 0.01)	1 (ref)	1 (ref)	1 (ref)	1 (ref)	1 (ref)	1 (ref)	1 (ref)	1 (ref)
Q2	(0.01, 0.06)	1.22 (0.61, 2.43)	1.49 (0.68, 3.27)	1.08 (0.45, 2.59)	1.49 (0.50, 4.48)	1.57 (0.87, 2.83)	1.82 (0.89, 3.72)	1.25 (0.64, 2.44)	1.71 (0.78, 3.73)
Q3	(0.06, 0.30)	1.02 (0.51, 2.03)	1.29 (0.61, 2.74)	0.79 (0.33, 1.90)	1.01 (0.37, 2.78)	1.38 (0.74, 2.57)	1.58 (0.77, 3.24)	1.11 (0.56, 2.21)	1.38 (0.64, 2.99)
Q4	(0.31, 1.24)	1.43 (0.73, 2.79)	1.98 (0.97, 4.02)	1.05 (0.33, 3.34)	2.27 (0.61, 8.44)	2.25 (1.19, 4.26)	3.03 (1.44, 6.38)	1.68 (0.87, 3.24)	2.20 (1.06, 4.56)
Q5	(1.24, 23.15)	1.15 (0.55, 2.39)	1.23 (0.56, 2.67)	1.12 (0.34, 3.67)	1.86 (0.49, 7.09)	1.53 (0.79, 2.98)	1.75 (0.80, 3.81)	1.38 (0.64,2.97)	1.54 (0.68, 3.47)
*P* trend		0.752	0.317	0.925	0.644	0.133	0.049	0.532	0.262
β-Carotene (mg/day)									
Continuous (1 mg/day intake increment)		1.00 (0.98, 1.02)	1.01 (0.98, 1.03)	1.01 (0.98, 1.04)	0.99 (0.96, 1.03)	1.00 (0.98, 1.02)	0.99 (0.96, 1.02)	0.98 (0.96, 1.00)	0.98 (0.95, 1.01)
Q1	(0.0009, 0.70)	1 (ref)	1 (ref)	1 (ref)	1 (ref)	1 (ref)	1 (ref)	1 (ref)	1 (ref)
Q2	(0.71, 1.88)	0.79 (0.39, 1.60)	0.64 (0.30, 1.38)	1.40 (0.54, 3.65)	1.43 (0.49, 4.12)	0.34 (0.18, 0.63)	0.36 (0.18, 0.73)	0.41 (0.21, 0.83)	0.30 (0.15, 0.63)
Q3	(1.90, 3.50)	1.11 (0.53, 2.31)	1.27 (0.55, 2.92)	0.91 (0.27, 3.02)	0.81 (0.22, 2.97)	0.29 (0.15, 0.58)	0.29 (0.14, 0.64)	0.62 (0.29, 1.36)	0.50 (0.21, 1.19)
Q4	(3.50, 7.19)	0.54 (0.26, 1.10)	0.59 (0.27, 1.30)	0.84 (0.27, 2.58)	0.71 (0.21, 2.40)	0.38 (0.18, 0.80)	0.47 (0.20, 1.12)	0.39 (0.17, 0.86)	0.39 (0.16, 0.98)
Q5	(7.20, 87.12)	0.84 (0.42, 1.70)	0.91 (0.40, 2.06)	0.91 (0.27, 3.02)	0.60 (0.16, 2.33)	0.36 (0.18, 0.70)	0.35 (0.16, 0.79)	0.41 (0.19, 0.90)	0.37 (0.15, 0.93)
*P* trend		0.086	0.079	0.740	0.547	0.003	0.009	0.068	0.025
β-Cryptoxanthin (mg/day)									
Continuous (1 mg/day intake increment)		0.97 (0.87, 1.08)	0.94 (0.83, 1.07)	0.75 (0.55, 1.03)	0.76 (0.59, 0.97)	0.88 (0.80, 0.96)	0.79 (0.70, 0.89)	0.92 (0.82, 1.02)	0.88 (0.78, 0.99)
Q1	(0.0003, 0.01)	1 (ref)	1 (ref)	1 (ref)	1 (ref)	1 (ref)	1 (ref)	1 (ref)	1 (ref)
Q2	(0.01, 0.04)	1.19 (0.62, 2.29)	1.21 (0.59, 2.48)	1.14 (0.50, 2.58)	1.37 (0.57, 3.28)	1.13 (0.65, 1.97)	1.05 (0.57, 1.92)	0.78 (0.40, 1.54)	0.82 (0.39, 1.74)
Q3	(0.04, 0.10)	1.30 (0.62, 2.72)	1.41 (0.62, 3.23)	1.24 (0.51, 3.06)	1.85 (0.68, 5.01)	1.00 (0.51, 1.95)	1.16 (0.55, 2.46)	1.14 (0.54, 2.43)	1.21 (0.52, 2.83)
Q4	(0.10, 0.59)	1.10 (0.53, 2.30)	1.12 (0.49, 2.57)	0.91 (0.36, 2.33)	1.02 (0.35, 2.98)	1.05 (0.54, 2.04)	1.17 (0.57, 2.44)	0.97 (0.48, 1.98)	0.98 (0.45, 2.13)
Q5	(0.60, 18.53)	1.11 (0.54, 2.27)	1.04 (0.45, 2.44)	0.53 (0.19, 1.48)	0.61 (0.22, 1.71)	0.68 (0.36, 1.29)	0.54 (0.25, 1.17)	0.71 (0.35, 1.42)	0.64 (0.29, 1.46)
*P* trend		0.960	0.895	0.628	0.385	0.525	0.215	0.512	0.483
Lutein + zeaxanthin (mg/day)									
Continuous (1mg/day intake increment)		0.99 (0.95, 1.02)	0.99 (0.95, 1.03)	1.01 (0.96, 1.05)	1.00 (0.95, 1.05)	0.99 (0.96, 1.03)	0.98 (0.94, 1.02)	0.98 (0.95, 1.02)	0.98 (0.94, 1.03)
Q1	(0.0001, 0.22)	1 (ref)	1 (ref)	1 (ref)	1 (ref)	1 (ref)	1 (ref)	1 (ref)	1 (ref)
Q2	(0.22, 0.63)	0.96 (0.45, 2.05)	0.95 (0.41, 2.20)	0.70 (0.24, 2.06)	0.64 (0.20, 2.06)	1.28 (0.69, 2.39)	1.15 (0.57, 2.31)	1.05 (0.53, 2.10)	1.22 (0.53, 2.80)
Q3	(0.63, 1.32)	1.29 (0.61, 2.74)	1.14 (0.48, 2.71)	0.81 (0.24, 2.77)	0.78 (0.19, 3.24)	1.56 (0.80, 3.02)	1.55 (0.73, 3.29)	1.27 (0.59, 2.75)	1.46 (0.61, 3.49)
Q4	(1.32, 3.20)	1.68 (0.80, 3.50)	1.54 (0.66, 3.58)	1.67 (0.62, 4.48)	1.34 (0.37, 4.86)	2.58 (1.27, 5.23)	1.94 (0.91, 4.13)	1.79 (0.80, 3.97)	1.61 (0.66, 3.95)
Q5	(3.20, 64.16)	0.92 (0.42, 2.01)	0.92 (0.37, 2.32)	1.21 (0.33, 4.38)	1.01 (0.20, 5.11)	1.45 (0.71, 2.98)	1.11 (0.49, 2.48)	1.14 (0.51, 2.51)	1.23 (0.47, 3.21)
*P* trend		0.234	0.513	0.288	0.577	0.100	0.338	0.557	0.824
Lycopene (mg/day)									
Continuous (1mg/day intake increment)		0.99 (0.96, 1.01)	1.00 (0.97, 1.03)	0.98 (0.95, 1.01)	1.00 (0.96, 1.03)	0.98 (0.97, 1.00)	0.98 (0.97, 1.00)	0.99 (0.98, 1.01)	1.00 (0.98, 1.02)
Q1	(0.0009, 0.001)	1 (ref)	1 (ref)	1 (ref)	1 (ref)	1 (ref)	1 (ref)	1 (ref)	1 (ref)
Q2	(0.001, 0.003)	0.79 (0.45, 1.37)	0.75 (0.40, 1.39)	0.90 (0.38, 2.10)	0.66 (0.26, 1.65)	0.96 (0.55, 1.70)	0.98 (0.53, 1.82)	0.95 (0.50, 1.81)	0.81 (0.39, 1.68)
Q3	(0.003, 0.006)	0.86 (0.45, 1.61)	0.96 (0.47, 1.94)	1.35 (0.54, 3.38)	0.87 (0.34, 2.23)	0.66 (0.36, 1.19)	0.68 (0.32, 1.42)	1.09 (0.54, 2.20)	0.93 (0.42, 2.07)
Q4	(0.006, 0.29)	0.79 (0.45, 1.40)	0.88 (0.45, 1.72)	1.85 (0.66, 5.15)	1.37 (0.48, 3.91)	0.83 (0.46, 1.51)	1.02 (0.50, 2.09)	1.00 (0.52, 1.94)	0.99 (0.45, 2.15)
Q5	(0.30, 124.49)	0.53 (0.29, 0.96)	0.71 (0.37, 1.37)	1.12 (0.38, 3.33)	1.08 (0.35, 3.31)	0.63 (0.36, 1.10)	0.80 (0.44, 1.44)	0.66 (0.35, 1.25)	0.87 (0.42, 1.78)
*P* trend		0.330	0.789	0.455	0.690	0.333	0.665	0.443	0.972

^1^ Odds ratios (and 95% confidence intervals) of the highest quantile compared to the lowest quantile of daily carotenoid intake on osteopenia bone mineral density were calculated by logistic regression analysis after adjusting for covariates. Model 1: adjusted for age and BMI. Model 2: adjusted for age, BMI, alcohol consumption, physical activity, education level, supplement use, energy-adjusted intake of fiber, vitamin C, calcium, and sodium, and serum 25(OH)D. BMI: body mass index.
